# Campylobacteriosis in Finland: Passive Surveillance in 2004–2021 and a Pilot Case-Control Study with Whole-Genome Sequencing in Summer 2022

**DOI:** 10.3390/microorganisms12010132

**Published:** 2024-01-09

**Authors:** Kristiina Suominen, Tessa Häkkänen, Jukka Ranta, Jukka Ollgren, Rauni Kivistö, Päivikki Perko-Mäkelä, Saara Salmenlinna, Ruska Rimhanen-Finne

**Affiliations:** 1 Department of Health Security, Finnish Institute for Health and Welfare, Mannerheimintie 166, 00271 Helsinki, Finland; tessa.hakkanen@thl.fi (T.H.); jukka.ollgren@thl.fi (J.O.); saara.salmenlinna@thl.fi (S.S.); ruska.rimhanen-finne@thl.fi (R.R.-F.); 2 Risk Assessment Unit, Finnish Food Authority, Mustialankatu 3, 00790 Helsinki, Finland; jukka.ranta@ruokavirasto.fi; 3 Department of Food Hygiene and Environmental Health, Faculty of Veterinary Medicine, University of Helsinki, Agnes Sjöbergin katu 2, 00790 Helsinki, Finland; rauni.kivisto@helsinki.fi; 4 Atria Plc, Itikanmäenkatu 3, 60100 Seinäjoki, Finland; paivikki.perko-makela@atria.com

**Keywords:** *Campylobacter* infections, demography, risk factors, whole-genome sequencing, source attribution, Finland, humans

## Abstract

Campylobacteriosis causes a significant disease burden in humans worldwide and is the most common type of zoonotic gastroenteritis in Finland. To identify infection sources for domestic *Campylobacter* infections, we analyzed *Campylobacter* case data from the Finnish Infectious Disease Register (FIDR) in 2004–2021 and outbreak data from the National Food- and Waterborne Outbreak Register (FWO Register) in 2010–2021, and conducted a pilot case-control study (256 cases and 756 controls) with source attribution and patient sample analysis using whole-genome sequencing (WGS) in July–August 2022. In the FIDR, 41% of the cases lacked information on travel history. Based on the case-control study, we estimated that of all cases, 39% were of domestic origin. Using WGS, 22 clusters of two or more cases were observed among 185 domestic cases, none of which were reported to the FWO register. Based on this case-control study and source attribution, poultry is an important source of campylobacteriosis in Finland. More extensive sampling and comparison of patient, food, animal, and environmental isolates is needed to estimate the significance of other sources. In Finland, campylobacteriosis is more often of domestic origin than FIDR notifications indicate. To identify the domestic cases, travel information should be included in the FIDR notification, and to improve outbreak detection, all domestic patient isolates should be sequenced.

## 1. Introduction

Campylobacteriosis is an important type of food- and waterborne gastroenteritis in humans, caused by the zoonotic *Campylobacter* bacteria. The most common species infecting humans are *Campylobacter jejuni* and *Campylobacter coli*, with *C. jejuni* causing almost 90% of reported cases [1,2]. The incubation period is usually 24–72 h, and the main symptoms are diarrhea, which may be watery or bloody, fever, and stomach cramps [3]. Symptoms usually pass within one week, although relapses may occur in 10–25% of cases [4]. Especially in immunocompromised patients, bacteremia and septicemia may develop [3]. Rare post-infection complications include Guillain-Barré syndrome, Reiter’s syndrome, and irritable bowel syndrome (IBS) [5]. In the EU, the annual number of campylobacteriosis cases has been estimated to be nine million, the disease burden of campylobacteriosis and its sequelae are 0.35 million disability-adjusted life years (DALYs) per year, and the annual cost is EUR 2.4 billion [6].

*Campylobacters* can be found in the digestive tracts of many animals [2], as well as in environmental waters [5]. Humans usually become infected via the fecal-oral route by eating or handling raw or undercooked chicken meat, ingesting unpasteurized milk, untreated water, or unwashed vegetables, or by direct contact with animals [2,4,5].

In the EU, campylobacteriosis has been the most common cause of human foodborne gastroenteritis since 2007 [1]. *Campylobacter* is commonly resistant to fluoroquinolones, which have been considered critically important antibiotics in severe infections [7]. In 2021 in the EU, *Campylobacter* was the fourth most common cause of foodborne outbreaks after *Salmonella*, Norovirus, and bacterial toxins, with 249 reported outbreaks caused by, among other things, broiler and bovine meat and meat products [1]. Further, in Finland, *Campylobacters* are the most common zoonotic bacteria which cause gastroenteritis. To target control measures, more detailed information on the sources of *Campylobacter* infection in Finland is needed.

In this study, we described the *Campylobacter* cases notified to the Finnish Infectious Disease Register (FIDR) in 2004–2021 and investigated registered food- and waterborne outbreaks caused by *Campylobacter* in 2010–2021. To identify risk factors for domestic *C. jejuni* infections, and to evaluate the role of different reservoirs in disease transmission, a pilot case-control study and source attribution were performed, and patient samples were analyzed using whole-genome sequencing (WGS).

## 2. Materials and Methods

### 2.1. Campylobacter Surveillance

Clinical microbiological laboratories in Finland are obliged to notify all laboratory-confirmed *Campylobacter* infections to the FIDR [8]. We formed a database of the case notifications from the FIDR between 1 January 2004 and 31 December 2021. We described the cases by gender, age, hospital district, and travel history, and calculated the incidence rate ratios (IRR) with 95% confidence intervals (CI) for gender, 5-year age groups, and hospital districts. From Statistics Finland, we retrieved annual data on Finn’s travels abroad to different destination regions in 2012–2021 [9]. The regions, as available from Statistics Finland, were Nordic countries, Russia and Baltic countries, Eastern and Western Europe, Southern Europe and Eastern Mediterranean, Americas, Africa, and Asia and Oceania. For Africa, the travel data were missing for 2013–2018. In 2012, there were 110,000 trips to Africa, and in 2019 there were 120,000. We estimated the annual travel numbers for 2013–2018 to be the mean of the years 2012 and 2019 (115,000 trips). For 2020–2021, the travel data were missing for the Americas and Africa, and for Asia and Oceania, the information was missing for 2021. We calculated incidences for cases who traveled to different regions of the world (cases/100,000 trips), and IRRs with 95% CIs for different destination regions. The IRRs were calculated using STATA 18.0 software (StataCorp LLC, College Station, TX, USA).

### 2.2. Outbreaks

An outbreak was defined as two or more temporally and/or geographically linked cases of campylobacteriosis. Since 2010, health officials in Finland have been obliged to report suspected food- and waterborne outbreaks to the National Online Food- and Waterborne Outbreak Register (FWO Register) and to write a report on the outbreak investigation and its results [10]. The notification is mandatory for outbreaks in facilities (for example in a school, a hospital, or a day-care center), if more than five non-family members fall ill, if the suspected source is a commercial product, if an outbreak in a restaurant is suspected to be caused by a widespread product, or if an exceptionally severe disease is diagnosed (for example botulism). We retrieved information from 1 January 2010 to 31 December 2021 of the notified food- and waterborne outbreaks with *Campylobacter* as the causative agent and described them by people falling ill and the source of the outbreak.

### 2.3. Case-Control Study

A case-control study was focused on risk factors of *C. jejuni* infections since *C. jejuni* causes most of the *Campylobacter* infections in Finland. This study was conducted as a pilot study over two months. A case was defined as a person with a laboratory-confirmed *C. jejuni* infection of domestic or unknown origin notified to the FIDR between 1 July 2022 and 31 August 2022. New cases were retrieved from the FIDR weekly. For every case, six controls matched by age, gender, and hospital district were chosen from the Population Information System. After retrieval, the cases and controls were sent an invitation letter to answer a questionnaire on the Internet. For cases and controls who had not answered within one week, a reminder letter was sent. The questionnaire could be completed in Finnish or Swedish, and contained questions about demographics and the clinical course of the disease, as well as animal, food, and environmental exposures. For the exposures, the cases were asked to consider the two-week period before falling ill, and controls were similarly asked to consider the two-week period before answering the questionnaire.

For statistical analyses, STATA 18.0 software (StataCorp LLC, College Station, TX, USA) was used. For the exposures, a univariate analysis was conducted with logistic regression to calculate odds ratios (OR) with 95% CIs. The match between cases and controls was broken for the analysis since controls could not be recruited for 26% of cases. Exposures with a *p*-value < 0.10 in the univariate analysis were included in the multivariable analysis. The exposures with 8% or more missing values were omitted. For the multivariable analysis, logistic regression with backward stepwise selection was used. The exposures were first excluded based on *p*-values, and the Akaike information criterion was used for selecting the final model [11].

### 2.4. Microbiological Samples and Methods

#### 2.4.1. Sample Collection and Handling

*Campylobacter* isolates from patient samples were sent to the bacteriology laboratory of the Finnish Institute for Health and Welfare (THL) from clinical microbiology laboratories across Finland during July and August 2022. The isolates sent included domestic *C. jejuni* isolated from patients sampled during the two-month period. In addition, samples from infections of an unknown origin or lacking a species-level identification were also collected. The received strains were combined with FIDR and questionnaire data, and samples from 185 cases who answered the questionnaire and had not traveled abroad within the two weeks prior to the onset of symptoms were selected for WGS. In addition, 47 *C. jejuni* isolates from animal and food samples were received from the Finnish Food Authority. The samples were taken between June and August 2022. Altogether 42 (89%) isolates originated from domestic poultry, 33 domestic broiler isolates were collected as part of national *Campylobacter* surveillance performed at slaughterhouses [12], four broiler and two turkey isolates were collected as part of voluntary industry sampling, and three broiler isolates were collected according to Microbiological Criteria Regulation [13,14]. Additionally, two isolates (4.3%) were obtained from imported broiler meat via their own-check activities, and three isolates (6.4%) were from ill fur animals: two from mink, and one from a raccoon dog. All received strains were grown on *Campylobacter* selective agar and on sheep blood agar in microaerobic conditions at 37–42 °C for 24–48 h.

#### 2.4.2. Whole Genome Sequencing (WGS) and Sequence Analysis

Altogether 185 patient isolates and 47 animal and food isolates were sequenced. DNA extraction and next-generation sequencing were largely carried out by LGC Genomic GMbH (Berlin, Germany) with Illumina paired-end technology (2 × 150 bp) on NovaSeq6000 (Illumina, San Diego, CA, USA) or the NextSeq (Illumina, San Diego, CA, USA) devices. Alternatively, all animal strains, and a few human sample repeats, were extracted and sequenced in-house. Extraction was performed with an Analytik Jena InnuPure C19 touch automated nucleic acid extraction machine (Analytik Jena, Jena, Germany) and a smart DNA prep (a) kit, while the sequencing was carried out with Illumina paired-end technology (2 × 150 bp) using the Illumina MiSeq sequencing machine and the Nextera XT Library Preparation kit (Illumina, San Diego, CA, USA).

An in-house pipeline was used for species verification and multilocus sequence typing (MLST). The Kraken2 software (version 1.0.0) and Bracken companion tool [15] were used for species identification, while MLSTs and clonal complexes (CCs) were derived from the pubMLST database [16] using stringMLST tool version 0.6.3 [17].

The Ridom SeqSphere program (version 8.5.1), with Velvet short read assembler (version 1.2.10), was used for genome assembly [18], as well as for core genome MLST (cgMLST) analysis and clustering. The clustering was performed with an in-house schema of 1135 core loci, based on the *C. jejuni* 4031 reference genome sequence (GenBank accession NC_022529.1), and clusters were defined as 99.41% allele similarity or higher [19]. This was the equivalent of less than seven allele differences.

The nucleotide sequence data reported in this study is available in the European Nucleotide Archive (ENA) at EMBL-EBI under the accession number PRJEB66857 (https://www.ebi.ac.uk/ena/browser/view/PRJEB66857, uploaded on 6 October 2023).

### 2.5. Source Attribution

The source attribution of the 185 domestic human isolates was studied using previously published MLST-typed isolates sampled from cattle (*n* = 102), broilers (*n* = 331), turkey (*n* = 32), and natural swimming waters (*n* = 30) [20]. The source attribution model corresponds to a supervised classification scheme where the source samples are used as training data to estimate the allele frequencies in the sources (as Dirichlet-posterior distributions, based on multinomial sample data). Consequently, the human isolates were probabilistically classified into the sources, based on their allele types and the allele type frequencies in the sources, and then the most probable source for each isolate was calculated. Additionally, the overall generalized source proportions were estimated as posterior distributions.

The source attribution results were computed using MCMC methods in R and OpenBUGS, based on the posterior distribution of the allele frequencies (in 7 loci) in four groups defined as source populations and the classification of human isolates into those groups (see Appendix A).

### 2.6. Ethical Approval Statement

The investigation was mandated by the Finnish Communicable Diseases Act 1227/2016 [8].

## 3. Results

### 3.1. Campylobacter Surveillance Data from Finnish Infectious Disease Register, 2004–2021

**Figure 1:**
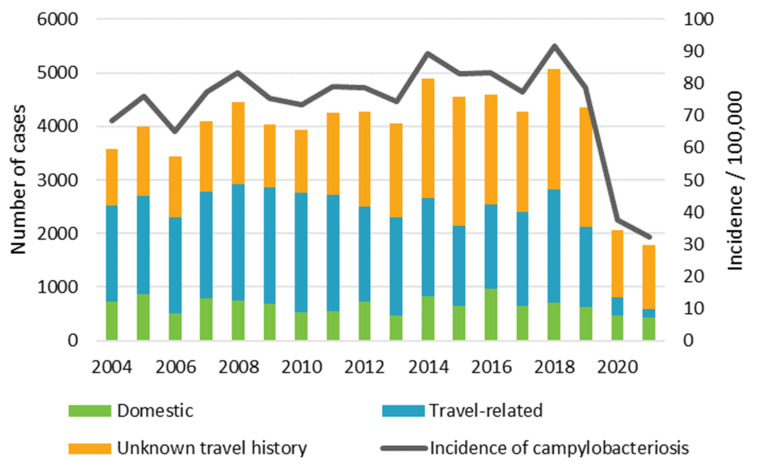
Number of *Campylobacter* cases according to travel history, and annual incidence per 100,000 people in Finland in 2004–2021.

**Figure 2:**
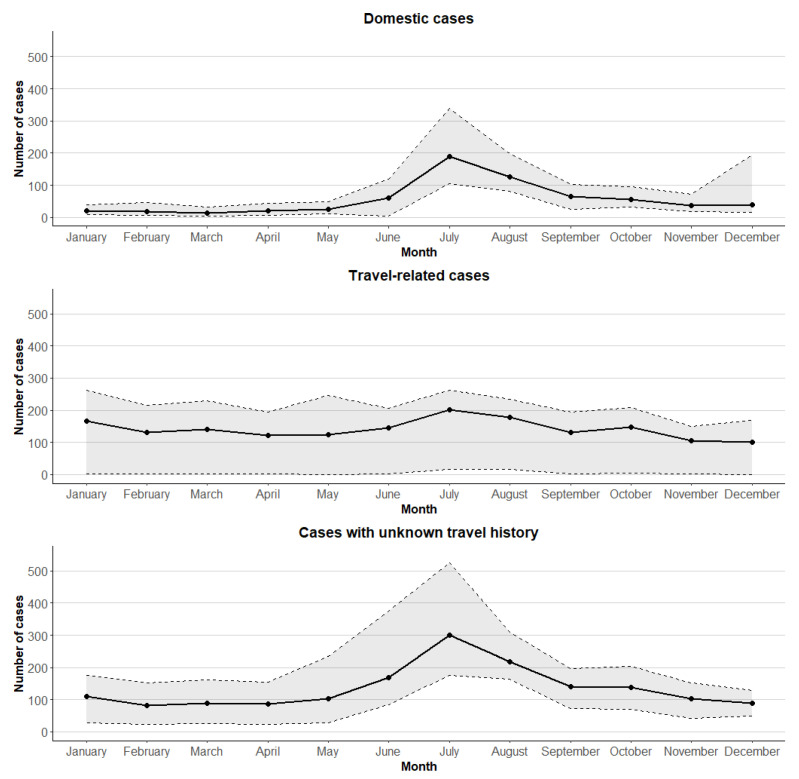
The mean monthly number of *Campylobacter* cases according to travel history in Finland in 2004–2021 (solid line). The minimum and maximum case numbers in the study period are shown with dashed lines, and the grey area represents the range of variation.

In total, 71,716 campylobacteriosis cases were notified to the FIDR during 2004–2021, of which 17% (11,996) were domestic, 42% (30,433) were travel-related, and 41% (29,287) were of an unknown origin (Figure 1). In 2020 and 2021 the case numbers dropped dramatically. The mean monthly number of domestic cases and cases with an unknown travel history peaked in July and August, whereas the number of travel-related cases was stable throughout the year (Figure 2). *C. jejuni* caused most of the infections (77%; 55,136), followed by *C. coli* (6%; 4120). For 17% of the cases (12,305) the species was not determined.

**Figure 3:**
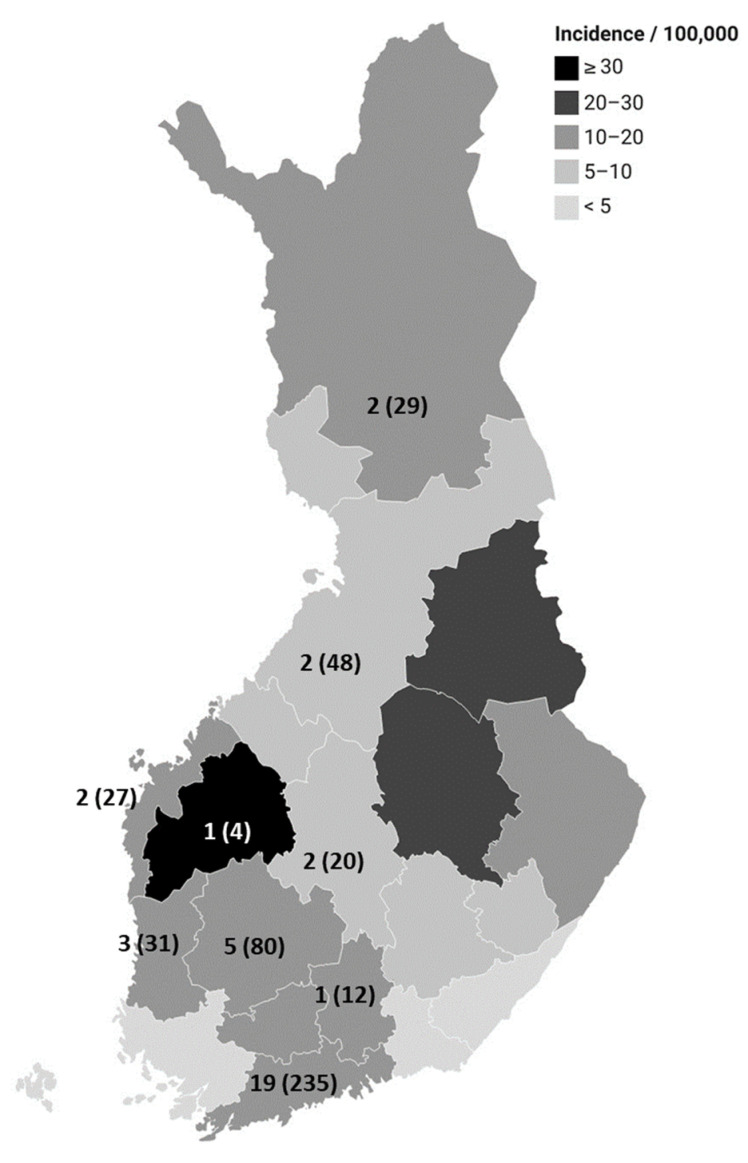
Incidence of domestic *Campylobacter* infections according to hospital district in Finland in 2004–2021. The numbers represent the food- and waterborne outbreaks caused by *Campylobacter* spp. in 2010–2021, and the number of people who fell ill in these outbreaks is presented in brackets.

The median age was 43 years (<1–99 years) for domestic cases, and 38 years (<1–99 years) for travel-related cases. In domestic cases, the incidence was the lowest for 5–14-year-olds (4.0/100,000), and highest for 20–54-year-olds (15.6/100,000). Of the cases, 59% (7045/11,996) and 52% (15,952/30,433) were male in domestic and travel-related cases, respectively. The incidence was 1.5-fold for domestic male cases when compared to domestic female cases (IRR 1.5, 95% CI 1.4–1.5, *p*-value < 0.001). Domestic cases were reported from all parts of Finland (Figure 3). When compared to other hospital districts, the incidences were highest in the South Ostrobothnia (IRR 2.6, 95% CI 2.5–2.8, *p*-value < 0.001) and North Savo (IRR 2.1, 95% CI 1.9–2.2, *p*-value < 0.001) hospital districts. The same hospital districts had the lowest proportion of cases with unknown travel histories (9% and 13%, respectively).

Of the domestic cases, 0.1% (12/11,996) died within 30 days of being tested (median age 84 years, range 22–94 years).

**Figure 4:**
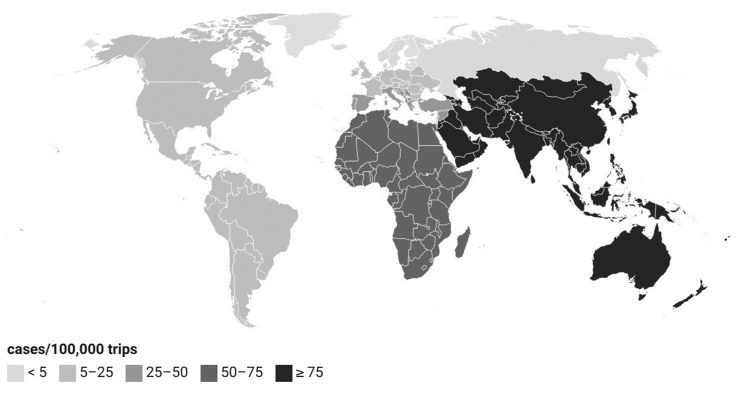
Incidence of Finland’s travel-related *Campylobacter* infections according to geographical region in 2012–2021. Regions: Nordic countries; Russia and Baltic countries; Eastern and Western Europe; Southern Europe and Eastern Mediterranean; Americas, Africa, Asia and Oceania. Data for the Americas and Africa available in 2012–2019, and for Asia and Oceania in 2012–2020.

Most travel-related infections originated from Southern Europe (24%, 7204/30,433) and Southeast Asia (21%, 6394/30,433). By country, most infections originated in Thailand (17%, 5092/30,433), Spain (13%, 3981/30,433), and Turkey (9%, 2877/30,433). In relation to the number of trips, the incidence was 9-fold for people who traveled to Asia and Oceania in 2012–2020, and 4-fold for people who traveled to Africa in 2012–2019 when compared to people who traveled to another region (IRR 9.0, 95% CI 8.6–9.3, *p*-value < 0.001, and IRR 4.1, 95% CI 3.8–4.5, *p*-value < 0.001, respectively; Figure 4).

### 3.2. Food- and Waterborne Outbreaks Caused by Campylobacter, 2010–2021

**Figure 5:**
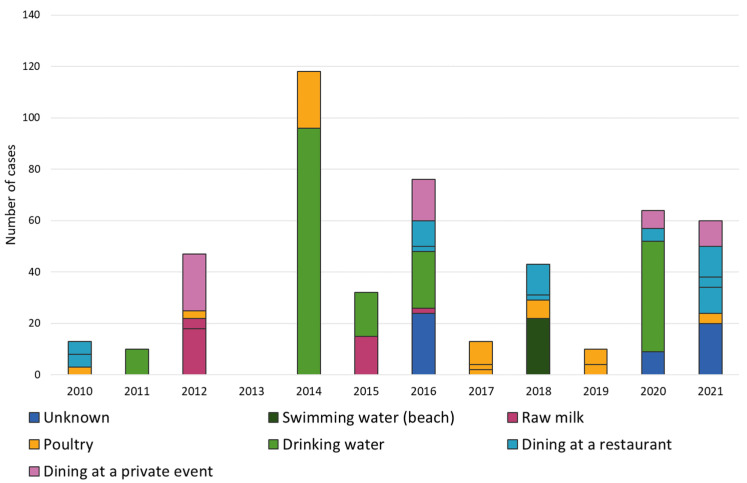
Number of food- and waterborne outbreaks caused by *Campylobacter* spp. by year, source, and number of cases in Finland in 2010–2021. Outbreaks are separated by lines, and sources are represented by different colors.

During 2010–2021, 31 foodborne and six waterborne outbreaks caused by *Campylobacter* were reported to the FWO register (Figure 3 and Figure 5). In the foodborne outbreaks, 276 people fell ill (mean 9 people, range 2–24). For 14 outbreaks, the reported source was dining, and for three outbreaks the source was unknown. Of the remaining 14 outbreaks, 10 were caused by poultry meat (chicken (5), duck breast (4), and pigeon (1)) and the rest (4) by raw milk. Of the six waterborne outbreaks, five were caused by drinking water, affecting 188 people (mean 38 people, range 10–96), and one originated from swimming water with 22 cases. The drinking water outbreaks originated from contaminated well water and water towers, and a fault in water treatment at a municipal waterworks. In the outbreaks, the strength of evidence varied from strong to weak.

### 3.3. Case-Control Study

**Figure 6:**
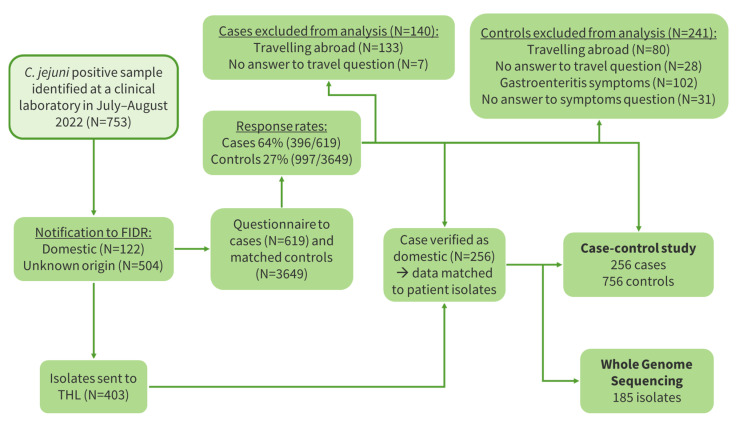
Flowchart of the *Campylobacter jejuni* case-control study protocol, Finland 2022.

**Figure 7:**
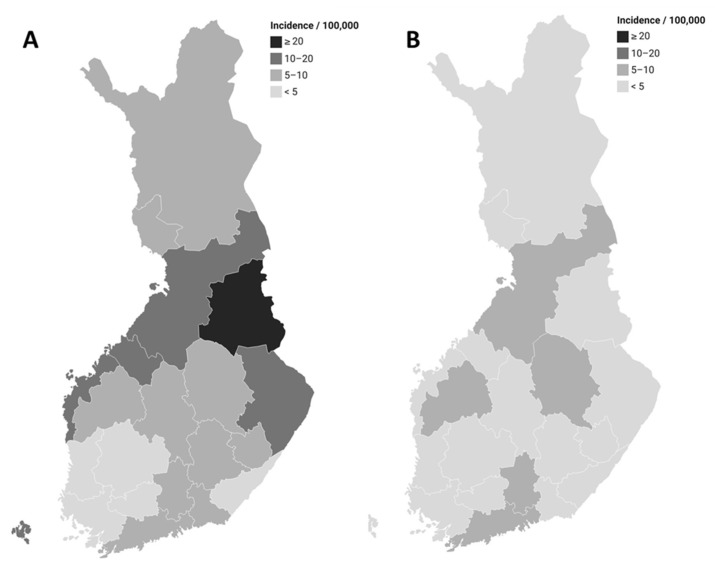
Incidence of *Campylobacter jejuni* infections (domestic and with unknown travel history) according to hospital district in Finland in July (**A**) and August (**B**) 2022.

In July–August 2022, 753 *C. jejuni* cases were notified to the FIDR, of which 626 were domestic or of an unknown origin (Figure 6). Their median age was 48 years (<1–96 years), and 56% (350/626) of them were male. Cases were reported from all parts of Finland with the highest incidence in the Kainuu Hospital District in July (Figure 7). The median duration from fecal sampling until notification was 6 days (1–19 days).

Addresses were obtained and invitation letters were sent to 619 cases and 3649 controls (Figure 6). The response rates were 64% and 27% for the cases and controls, respectively. The exclusion criteria for the cases and controls are shown in Figure 6. Of the cases who answered the questionnaire, 79% (314/396) had an unknown travel history according to the FIDR. Of them, 55% (174/314) were of domestic origin. Based on this, we estimated that of all cases reported to FIDR, 39% were of domestic origin and 61% were travel-related.

The median age of the cases was 54 years (<1–92 years) and of the controls it was 51 years (<1–92 years). Of the 256 cases and 756 controls, 143 (56%) and 393 (52%) were male, respectively. The mean duration of cases’ symptoms was 10 days (2–54 days), and the most common symptoms reported were diarrhea (92%), fever (71%), and abdominal pain (61%). Bloody diarrhea was reported by 14%. The median duration from the onset of symptoms until fecal sampling was 4 days (<1–68 days). Of the cases, 42% (107/256) received intravenous fluids and 30% (78/256) were hospitalized for at least one night. The mean duration of hospitalization was 3 nights (1–13 nights).

Several exposures were associated with *C. jejuni* infection in the univariate analysis (Table S1). In the multivariable analysis, *C. jejuni* infection was associated with eating undercooked broiler or turkey meat, using medication to treat gastric acidity, swimming in natural waters or drinking untreated water, eating minced beef, having contact with wild birds or their feces, and handling animal feces or manure (Table 1).

### 3.4. Cluster Analysis of Bacterial Isolates

**Figure 8:**
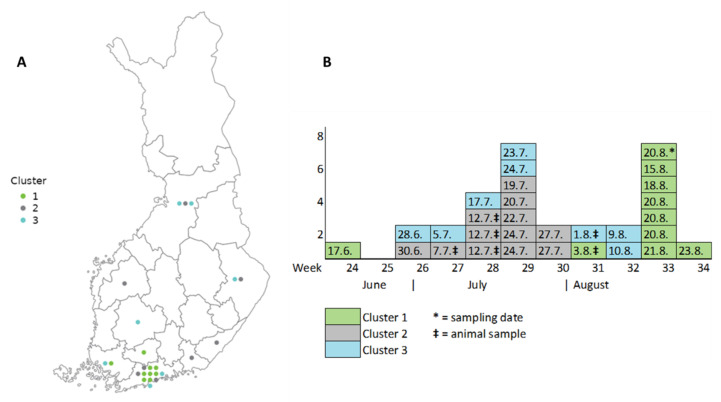
The three largest *Campylobacter jejuni* clusters according to hospital district (**A**) and the cases’ symptom onset date and sampling date of animal samples (**B**) in Finland, July–August 2022. For one case the symptom onset date is unknown, and the sampling date is provided instead.

The clinical microbiology laboratories sent 403 *C. jejuni* isolates from 392 cases to the THL. Of the 185 sequenced strains from domestic cases, 34 different MLSTs were identified. Altogether, 22 cgMLST clusters with two or more patient isolates were identified, containing 37% (68/185) of the *C. jejuni* isolates (Table 2). Seven clusters contained animal or food isolates, mainly from poultry. Three clusters with five or more cases were identified and were caused by ST-50, ST-230, and ST-45 (with 9, 8, and 7 cases, respectively). Similar MLSTs were also found in broiler meat samples and the cases reported the consumption of broiler meat in the questionnaire study. The cases in the ST-50 cluster were situated mostly in the Helsinki and Uusimaa hospital district, and the symptoms started within one week, except for one case (Figure 8). The broiler sample associated with this cluster was taken two weeks prior to the sampling dates of eight temporally clustered cases. The clustered cases in ST-230 and ST-45 were from different parts of Finland (Figure 8). Similar to the ST-50 cluster, the symptom onset of cases in ST-230 was within one week, except for one case, and the four broiler samples were taken one to two weeks prior to the sampling dates of seven temporally clustered cases. The symptom onset of cases in the ST-45 cluster was spread over seven weeks, and the positive broiler sample associated with this cluster was taken after the sampling dates of five of the human cases. None of the clusters were reported to the FWO register.

### 3.5. Source Attribution

**Figure 9:**
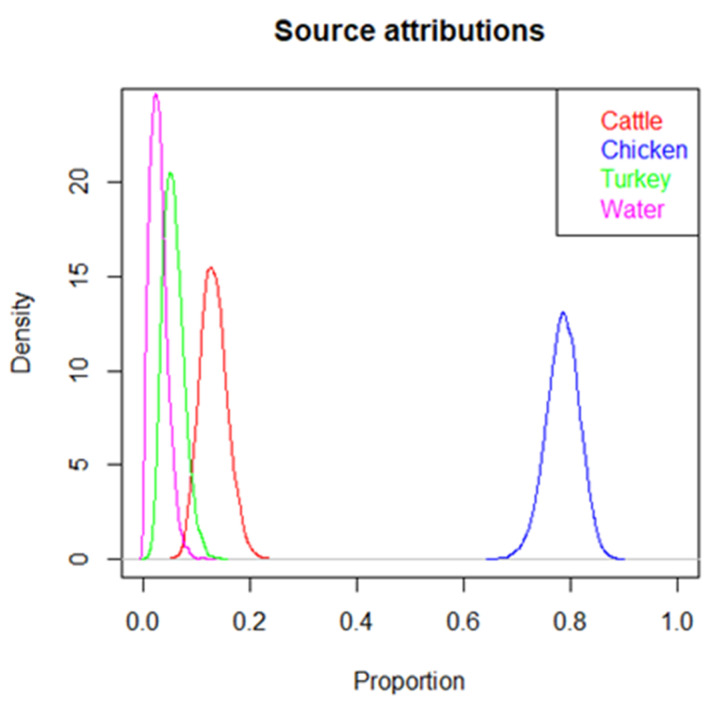
Estimated population proportions of Finnish human campylobacteriosis cases attributable to four sources (cattle, chicken, turkey, water), based on the probabilistic classification of the sampled 185 cases. Previously published MLST data from animal, food, and environmental isolates from Finland were used for source attribution analysis [20].

The general population attributable fractions and 95% credible intervals were 78% (71–84%) for broiler chicken, 13% (8–18%) for cattle, 5% (2–10%) for turkey, and 2% (0–6%) for water (Figure 9). Of the 185 case samples, 147 were most probably attributed to broiler, 24 to cattle, nine to turkey, and five to water. Of the 140 human cases who had reported eating poultry meat, 116 (83%) had broiler (110) or turkey (6) as the most probable source according to the source attribution model. Of the 40 human cases who had reported not eating poultry, 35 (88%) had broiler (32) or turkey (3) as the most probable source. Of the 148 human cases who had reported eating beef, 21 (14%) had cattle as the most probable source according to the source attribution model. Of the 28 human cases reporting not eating beef, three (11%) had cattle as the most probable source.

## 4. Discussion

In Finland, *Campylobacter* is the most common cause of bacterial gastroenteritis. From 2004 to 2019, the incidence underwent a slightly increasing trend, while case numbers dropped dramatically in 2020 and 2021. A similar reduction in campylobacteriosis case numbers has also been observed elsewhere and can be explained by restrictions on travel, public events, and restaurants, as well as improved hygienic practices due to the COVID-19 pandemic [1,21].

Almost half of the campylobacteriosis case notifications to the FIDR lacked information on travel history. In our case-control study, more than half of the cases with an unknown travel history in the FIDR reported not traveling abroad. Further, the numbers of both domestic cases and cases with an unknown travel history peaked in July and August. Considering these findings, we estimated that two-fifths of all cases could be domestically acquired, indicating that more cases than previously considered, based on FIDR notifications, are of domestic origin.

We identified 22 clusters covering one-third of the 185 domestic cases’ samples. Three larger clusters with seven to nine cases were identified. None of the clusters detected were notified to the FWO register, indicating that many smaller, widespread, or prolonged *Campylobacter* outbreaks go undetected. Without comprehensive surveillance, widespread outbreaks may be difficult to detect since they often are indistinguishable from sporadic cases [22]. In Denmark and Sweden, campylobacteriosis clusters have been identified using WGS [23,24]. In Finland, several listeriosis outbreaks have been detected since the introduction of WGS in routine surveillance [25]. To improve outbreak detection, we recommend that all domestic *Campylobacter* patient isolates should be sequenced. When retrieving cases from the FIDR in the case-control study, we noticed that some of them were reported after a prolonged period of time. The median duration from the onset of symptoms to fecal sampling was four days, and from fecal sampling to notification it was six days. However, the longest period from fecal sampling to notification was almost three weeks. A delay in fecal sampling and/or notification affects the detection and investigation of outbreaks.

Of the cases with a known hospitalization status, 23% were hospitalized in the EU in 2021 [1]. In our case-control study, one-third of the cases were hospitalized, almost half reported receiving intravenous fluids, and one-sixth had bloody diarrhea. These findings are in line with a previous Finnish study during the seasonal peak in 2002 [26]. Although it is probable that people with more severe diseases were more prone to answer the questionnaire, our study highlights the severity of campylobacteriosis. The mortality rate in Finland in 2004–2021 was slightly higher than the mortality rate in the EU in 2021 [1]. In Denmark and the Netherlands, campylobacteriosis has been estimated to cause the highest burden of disease of many foodborne pathogens, with 1709 DALYs in 2017 in Denmark, and 3300 DALYs in 2019 in the Netherlands [27,28]. In Finland, the sequelae of campylobacteriosis are not reported, and the disease burden has not been estimated. Considering the high case numbers, the severity of the disease, and the potential sequelae such as reactive arthritis, Guillain-Barré syndrome, and IBS, the burden of campylobacteriosis can be considered also significant in Finland.

In our study, several findings were in line with previous studies: a summer peak was observed in the number of domestic cases, campylobacteriosis was more common in males than in females, and the exposures associated with *C. jejuni* infections in the case-control study were similar (eating undercooked broiler or turkey meat, using medication to treat gastric acidity, swimming in natural waters or drinking untreated water, eating minced beef, having contact with wild birds or their feces, and handling animal feces or manure). A peak during the summer months is seen also in other EU countries [1,29]. The incidence for males has been higher than for females in other Nordic countries as well as in the EU in general [1,29]. Green et al. observed that males already have higher incidences from infancy [30]. According to them, this suggests that the difference is not only due to behavioral factors but at least in part to physiological or genetic factors, such as sex hormones or immune responses. In Finland, from 2004 to 2021, incidences of domestic cases were the lowest for 5–14-year-olds and the highest for 20–54-year-olds. We did not observe a clear peak in any one age group. In other Nordic countries in 2000–2015, the highest incidences were for 0–4-year-olds and 20–29-year-olds [29], and in the EU in 2021, also for 0–4-year-olds [1]. The reasons for the lack of a peak for small children in Finland are unknown but could be explained by better hygienic practices or being exposed differently than in other countries (for example having less contact with animals) [29]. The incidence of domestic campylobacteriosis was the highest in the South Ostrobothnia and North Savo hospital districts compared to the rest of Finland. No large outbreaks were reported in these areas. This finding may be at least partially explained by the same hospital districts having the lowest proportion of cases with missing travel information. Additionally, a large proportion of Finnish broiler farms are situated in the region of South Ostrobothnia [31]. Previously, it has been shown that residential proximity to poultry farms was associated with campylobacteriosis [32]. In the regions of North Savo and Ostrobothnia, the densities of cattle are high [33].

In 2010–2021, foodborne outbreaks caused by *Campylobacter* were small, and in many of them, a source with microbiological evidence was not found. The outbreaks with identified sources were caused by poultry meat and raw milk. Similarly, in Denmark in 2005–2015, most outbreaks were small and often involved chicken when a likely source could be identified [34]. Chicken and dishes containing chicken liver have been implicated in foodborne outbreaks in many countries worldwide [21]. In the USA, outbreaks have been commonly caused by dairy products, followed by chicken and vegetables [35]. Unpasteurized milk has also been implicated in outbreaks in Sweden and the UK [21]. In our case-control study, *C. jejuni* infections were strongly associated with eating undercooked broiler or turkey meat. Further, according to the source attribution model, broiler or turkey meat was the most likely source for most of the cases. The model, however, suggested broiler or turkey meat to be the most likely source also for the majority of cases who reported not eating poultry. No obvious association between the poultry consumption reported by the cases and the source attribution of the cases was seen. However, this could be explained by other poultry-related transmission, such as via the environment.

Since 2004 in Finland, every slaughter batch of broilers is examined for *Campylobacter* in June–October, and samples are taken in other months according to a separate plan [36]. In June–October 2022, 4.2% of examined flocks were *Campylobacter* positive, and during the rest of the year, 0.3% [36]. Previously, it has been estimated that one-fifth to one-third of domestic human cases in Finland might be related to consuming fresh chicken meat [20,37], even though the broiler flock prevalence is relatively low. In the EU, handling, preparing, and consuming broiler meat is estimated to cause 20–30% of human infections and around 50–80% of human campylobacteriosis cases might be attributed to the chicken reservoir in general, including by direct contact or via the environment [38]. However, since there is often much more data on poultry reservoirs than on other animals or the environment, the importance of poultry may be overestimated by MLST studies and source attribution. Additionally, other animals might be colonized by the poultry-associated strains. Nevertheless, it seems that poultry, and especially broiler meat, still remains an important source of campylobacteriosis in Finland. Information campaigns on campylobacteriosis and proper kitchen hygiene to avoid cross-contamination could be implemented especially during summer months, when *Campylobacters* are the most prevalent in both humans and broilers. During the summer, many Finns also prepare food outside by grilling and spend time at their summer cottages where hygienic practices might be more difficult to maintain. Furthermore, well water is often used for drinking at summer cottages, and swimming in surface waters is common. Both have previously been shown to be associated with the risk of *Campylobacter* infections in Finland [39].

In many studies, poultry is implicated as the most important source of human campylobacteriosis, followed by cattle [40,41]. Cattle have been thought to be a potentially significant source of campylobacteriosis also in Finland [42]. In addition to eating contaminated meat, campylobacteriosis can be contracted by drinking raw milk and by direct contact with animals [3]. In our study, 13% of cases were attributed to cattle in the source attribution, which is in line with previous studies [40,41]. In our case-control study, campylobacteriosis was also associated with eating minced beef. Minced beef has been linked to campylobacteriosis also in Denmark [43]. In 2011–2012, Llarena et al. investigated domestic retail minced beef samples in Finland for *C. jejuni* and found that none of the samples were culture-positive [44]. The survival of *Campylobacters* on bovine carcasses has also been shown to decrease during the chilling process due to drying of the carcass surface and exposure to atmospheric oxygen [45]. The potential transmission routes from cattle to humans or possible common sources need to be investigated further.

*Campylobacters* are often found in various water sources [46], and surface waters have been identified as an important environmental source for human infections [39,40]. Drinking untreated water and swimming in natural waters was also associated with campylobacteriosis in our study. In Finland, people may drink untreated water, for example, from the wells at summer cottages, or while hiking in nature. Well water should be tested at regular intervals, only treated water should be consumed in nature, and the ingestion of water while swimming should be avoided to prevent infections. During 2010–2021, *Campylobacters* caused a few waterborne outbreaks in Finland. *Campylobacters* have been the cause of many waterborne outbreaks worldwide, many of them affecting hundreds or thousands of people [46]. On average, more people are afflicted in waterborne outbreaks compared to foodborne ones. Heavy rainfall has been implicated to be the probable mechanism in many outbreaks [46]. In the Nordic countries, increases in campylobacteriosis cases have often been preceded by a rise in temperature and heavy rainfall [47]. Since climate change is expected to cause heavy rainfall in Finland more often, it is important to prepare for more campylobacteriosis cases and waterborne outbreaks. To avoid waterborne outbreaks, the entry of contaminated surface waters or sewage to water sources and distribution networks should be prevented.

Many domestic and wild animals and birds are known to harbor *Campylobacters* in their gastrointestinal tracts [2]. In Finland, *C. jejuni* has been isolated from several wild bird species (western jackdaws, mallard ducks, and pheasants with 43%, 76%, and 9% of the tested samples positive, respectively), among which some of the mallard duck and pheasant isolates were genetically very similar to human isolates [48]. Additionally, of the hunted game birds sampled, 27% of woodpigeons, 22% of pheasants, 71% of mallard ducks, and 73% of teals were found to be PCR positive for *Campylobacters* [49]. In addition to our study, contact with animal feces has also previously been identified as a risk factor for campylobacteriosis [43]. Wild birds and farm animals can also contaminate environmental waters [46]. It is important to wash hands thoroughly after handling animal or bird feces or carcasses and to prevent animal or bird feces from entering drinking water sources.

In our study, using medication to treat gastric acidity was associated with campylobacteriosis. A low gastric pH due to hydrochloric acid is considered an important natural defense against enteric pathogens [50]. Proton pump inhibitors (PPIs) are drugs used to treat conditions caused by stomach acidity. However, their use has been associated with campylobacteriosis in our study and in previous studies, for example in New Zealand [41]. A recent meta-analysis found a higher risk for PPI users of developing campylobacteriosis or other bacterial gastroenteritis compared to nonusers [51]. Since the use of PPIs has increased in recent decades [52], their potential adverse effects should be better communicated to the public.

In the Finnish surveillance data, the mean monthly number of travel-related cases remained stable throughout the year. In 2012–2021, travel-related campylobacteriosis originated most often from Southern Europe and Southeast Asia, and the incidence was highest for those traveling to the Asia and Oceania region compared to other regions defined by Statistics Finland. In the EU in 2021, most travel-related infections were acquired in Southern Europe (with 28% of cases originating from Spain) [1]. In Southeast Asia, on the other hand, *Campylobacter* is known to cause a larger proportion of traveler’s diarrhea cases compared to other regions of the world [53]. Our results are in line with these previous studies. The measures to prevent traveler’s diarrhea in general also apply to campylobacteriosis. To reduce travel-related *Campylobacter* infections, communication of the preventive measures to Finnish travelers could be improved.

### Limitations

A limitation of our study was the short study period of two months, due to which we could not detect any long-lasting outbreaks or seasonal differences in the exposures. In the source attribution, the source samples represented historical samples, whereas the human samples represented the summer of 2022. Therefore, the analysis relies on assuming some stability of the allele distributions in the source populations over time. Recall bias is always possible in case-control studies. To minimize its effect, the cases were asked to complete the questionnaire shortly after illness and questions were mostly asked about recurring or outstanding exposures that are easier to remember. In the surveillance data, a large proportion of notifications lacked information on the travel history, which may distort the demographics.

## 5. Conclusions

In Finland, *Campylobacter* infections are more often of domestic origin than FIDR notifications indicate. In our study, infections were often severe and one-third of the cases were hospitalized. Widespread or prolonged outbreaks go unnoticed without comprehensive microbiological surveillance. To correctly identify the domestic cases, information on travel history should be included in the FIDR notification, and to improve outbreak detection, all domestic patient isolates should be sequenced. Poultry, especially broiler meat, is an important source of campylobacteriosis in Finland. To better estimate the significance of other sources, more extensive sampling, and comparison of isolates with modern microbiological methods are needed. The communication of the preventive measures of campylobacteriosis could be improved.

## Figures and Tables

**Table 1:** The exposures associated with *Campylobacter jejuni* infection in multivariable analysis (*p* < 0.05).

Exposure	Odds Ratio (95% CI ^a^)	*p*-Value
Eating undercooked broiler or turkey meat	20 (6.0–69)	<0.001
Medication to treat gastric acidity	2.7 (1.6–4.5)	<0.001
Swimming in natural waters or drinking untreated water	2.4 (1.5–4.0)	<0.001
Eating minced beef	2.0 (1.1–3.5)	0.018
Contact with wild birds or their feces	2.8 (1.1–7.1)	0.028
Handling animal feces or manure	1.8 (1.1–3.2)	0.030

^a^ = confidence interval.

**Table 2:** *Campylobacter jejuni* clusters with two or more patient isolates by clonal complex and sequence type, July–August 2022, Finland.

MLST		No. of Clusters	Clusters: No. of Patient Isolates	Clusters: No. of Animal/Food Isolates	No. of Cases in the Cluster Who Had Eaten Broiler or Turkey Meat
Clonal Complex	Sequence Type (ST)				
ST-45 complex	45	7	7	1 broiler isolate	4/7
			4	1 turkey isolate	4/4
			3	1 broiler isolate	3/3
			3	0	
			2	5 broiler isolates	2/2
			2	0	
			2	0	
	230	1	8	4 broiler isolates	6/8
	11	1	2	0	
	1701	1	2	0	
	2406	1	2	0	
ST-677 complex	677	3	3	0	
			2	0	
			2	0	
	6514	2	2	0	
			2	0	
ST-21 complex	50	1	9	1 broiler isolate	9/9
	19	1	2	0	
ST-1287 complex	945	1	2	0	
ST-257 complex	2254	1	3	0	
ST-48 complex	918	1	2	1 mink strain	
does not belong to a clonal complex	2274	1	2	0	

## Data Availability

The raw/processed data analyzed in the study cannot be shared due to the European General Data Protection Regulation.

## Supplementary Materials

The following supporting information can be downloaded at: https://www.mdpi.com/article/10.3390/microorganisms12010132/s1, Table S1: The results of a univariate analysis of exposures associated with domestic *Campylobacter jejuni*-infection (*p* < 0.1).

## Appendix A

Source attribution model [54]:

Observed allele type counts (A) in each source (*s* = 1,…,4), at each locus (*j* = 1,…,7) were modeled as multinomially distributed:

$$P\left(A_{s,j,1},\ldots ,A_{s,j,n(j)}\;\right|{\;q}_{s,j,1},\ldots ,q_{s,j,n\left(j\right)},N_{s})=Multinomial({\;q}_{s,j,1},\ldots ,q_{s,j,n\left(j\right)},N_{s})$$

With *N_s_* as the number of sampled isolates from each source population s, and *n(j)* as the number of different allele types at *j*th locus in the whole data. With Dirichlet(1,1,…,1) prior, the posterior distribution of the unknown population frequencies of alleles becomes

$$P\left(q_{s,j,1},\ldots ,q_{s,j,n(j)}\;\right|A_{s,j,1},\ldots ,A_{s,j,n\left(j\right)},N_{s})=Dirichlet({\;A}_{s,j,1}+1,\ldots ,A_{s,j,n\left(j\right)}+1)$$

The uncertainty distribution of the allele frequencies *q* then depends only on the sample data from each source population. i.e., the samples of isolates from the sources were used for estimating source-specific allele frequencies. Conditionally on the unknown source *S_i_* for each human isolate, its allele type *H* at locus *j* had a multinomial distribution

$$P\left(H_{i,j,1},\ldots ,H_{i,j,n(j)}\;\right|\;S_{i}=s,\;q_{s})=Multinomial({\;q}_{s,j,1},\ldots ,q_{s,j,n\left(j\right)},1)$$

and the probability for the source was *P(S_i_ | p)* = Categorical(*p*), with prior *p* ~ Dirichlet(1,1,1,1). From these, the posterior probability for population source proportions *p* and case sample attributions *S*, as well as population allele frequencies *q* was constructed in MCMC simulations from two parts

(A1)
$$\;P\left(q\;|\;A,\;N\right)=\prod_{s=1,\ldots ,4}P\left(q_{s,j,1},\ldots ,q_{s,j,n(j)}\;\right|A_{s,j,1},\ldots ,A_{s,j,n\left(j\right)},N_{s})$$

and

(A2)
$$P(p,S|H,q)\propto \prod_{\begin{array}{c} i=1,\ldots ,185\\ j=1,\ldots ,7 \end{array}}P\left(H_{i,j,1},\ldots ,H_{i,j,n(j)}\;\right|\;S_{i},\;q_{S_{i}})P(S_{i}|p)P(p)$$

where the uncertain values of *q* are simulated from (Equation (A1)).

This makes a two-step classification model where in the first step the frequencies *q* are simulated from *P(q|A)* and the attributions *S* and source proportions *p*, given the *q* and *H* from *P(p,S|H,q)*. A fully connected one-step model for all *p, q, S* where the frequencies *q* would also be partly estimated accounting for the allocations of human isolates during simulation led to inconsistent results where the final source attributions differed between MCMC simulations and initial values used. The most probable source for each human isolate i was given as the source *S_i_* with the highest posterior probability. The population source attributions (in Figure 9) were given as the posterior distributions of *p*_1_,…, *p*_4_. The simulations were implemented both in OpenBUGS (using cut-functions for simulating *q* independently from human data) and also directly as Gibbs sampler in R (version 4.3.0) which gave the same results.
