# Supplementary material for: Campylobacteriosis in Finland: Passive Surveillance in 2004–2021 and a Pilot Case-Control Study with Whole-Genome Sequencing in Summer 2022
Source: Microorganisms. 2024 Jan 9;12(1):0. doi: 10.3390/microorganisms12010132 (PMC11154465; doi:10.3390/microorganisms12010132)
Supplement: Supplementary file 1 [file microorganisms-12-00132-s001.zip › microorganisms-2768188-supplementary.pdf]

Table S1. The results of a univariate analysis of exposures associated with domestic *Campylobacter jejuni* -infection ($p<0.1$).

Exposure agent	Cases (N=256) exposed % (n/N)	Controls (N=756) exposed % (n/N)	Odds ratio (OR) (95% CI [1])	p-value
Medication to treat gastric acidity	31 (76/244)	16 (116/728)	2.39 (1.68–3.38)	<0.001
Eating unheated berries	68 (167/245)	83 (622/751)	0.44 (0.32–0.63)	<0.001
Swimming in natural waters or drinking untreated water	53 (136/255)	39 (294/747)	1.76 (1.31–2.37)	<0.001
Eating undercooked broiler or turkey meat	10 (24/247)	1 (7/734)	11.18 (4.58–31.04)	<0.001
Eating soft cheeses made with unpasteurized milk	25 (58/228)	44 (322/731)	0.43 (0.31–0.61)	<0.001
Sorting and/or composting biowaste	59 (147/248)	71 (526/741)	0.59 (0.44–0.81)	0.001
Eating in a restaurant (also takeaway)	69 (172/250)	78 (587/749)	0.61 (0.44–0.85)	0.002
Noticed more flies than usual	3 (8/242)	9 (66/660)	0.34 (0.14–0.73)	0.003
Contact with wild birds or their feces	11 (27/251)	6 (42/751)	2.03 (1.18–3.46)	0.005
Eating fish and/or seafood	75 (182/242)	83 (615/744)	0.64 (0.44–0.92)	0.010
Grilling meat	50 (123/246)	41 (302/742)	1.46 (1.08–1.97)	0.011
Handling animal feces or manure	25 (57/225)	18 (130/726)	1.56 (1.07–2.25)	0.014
Family member having gastroenteritis	7 (17/250)	3 (24/738)	2.17 (1.07–4.29)	0.015
Eating homegrown vegetables	29 (71/248)	37 (275/746)	0.69 (0.49–0.95)	0.018
Eating minced beef	80 (194/244)	72 (537/742)	1.48 (1.03–2.15)	0.027
Gutting animals	4 (10/252)	2 (12/741)	2.51 (0.96–6.42)	0.029
Using cortisone medication	15 (37/240)	11 (78/727)	1.52 (0.96–2.35)	0.052
Eating raw sausage	7 (18/249)	12 (85/737)	0.60 (0.33–1.03)	0.055
Never using separate utensils for raw meat and vegetables	15 (35/233)	10 (74/706)	1.51 (0.95–2.37)	0.061
Regular animal contact	49 (124/254)	42 (318/751)	1.30 (0.97–1.75)	0.072
Living next to a production animal farm	19 (46/248)	14 (100/721)	1.41 (0.94–2.10)	0.076
Eating liver pâté	4 (10/249)	7 (52/738)	0.55 (0.25–1.12)	0.088

[1] = confidence interval
